# Effects of diluted seawater in drinking water on physiological responses, feeding, drinking patterns, and water balance in crossbred dairy goats

**DOI:** 10.14202/vetworld.2024.2398-2406

**Published:** 2024-10-31

**Authors:** Thiet Nguyen, Khang Van Truong, Khang Kim Thi Nguyen, Ngu Trong Nguyen, Narongsak Chaiyabutr, Sumpun Thammacharoen

**Affiliations:** 1 Department of Agricultural Technology, College of Rural Development, Can Tho University, Can Tho City, 94000, Vietnam; 2 Department of Animal Science, College of Agriculture, Can Tho University, Can Tho City, 94000, Vietnam; 3 Department of Physiology, Faculty of Veterinary Science, Chulalongkorn University, Bangkok 10330, Thailand; 4 The Academy of Science, The Royal Society of Thailand, Dusit, Bangkok 10300, Thailand

**Keywords:** antidiuretic hormone, dairy goat, kidney, saline water, water balance

## Abstract

**Background and Aim:**

In tropical regions, the intrusion of saline from seawater (SW) due to global warming and sea level rise in recent years is an important natural factor influencing goat well-being. This study aimed to determine the effects of diluted SW in drinking water on the physiological responses and eating and drinking patterns of crossbred dairy goats under tropical conditions.

**Materials and Methods:**

Twenty dairy goats were divided into four groups (five animals each) based on body weight and milk yield. Animals received either fresh drinking water (SW0.0, control) or diluted SW at concentrations of 0.5% (SW0.5, low salinity), 1% (SW1.0, moderate salinity), and 1.5% (SW1.5, high salinity). The experiment was performed for 49 days (1^st^–7^th^ week). Throughout this period, daily food and water intake were measured every day. In addition, blood collection was performed on day 25. Total urine and feces were collected from days 25 to 29. Meal and drinking patterns were determined on days 31 and 32.

**Results:**

Salinity did not influence dry matter intake throughout the experiment (p > 0.05). However, SW had a significant effect on eating patterns. The effect of SW on water intake (WI) was pronounced from the 2^nd^ to 7^th^ weeks of this experiment (p < 0.05). The water balance decreased and plasma antidiuretic hormone levels increased from SW1.5 to SW2.5 compared to the other treatments. Rectal temperature and respiration rate increased from 15:00 to 17:00 in SW1.5 patients. The concentrations of plasma electrolyte, creatinine, and heat shock protein 70 did not differ between treatments (p > 0.05). The urinary excretion of Na^+^ from SW1.5 and K^+^ and Cl^-^ from SW1.0 was higher than that from SW0.0 and SW0.5 (p < 0.01).

**Conclusion:**

Lactating crossbred goats adapted to low and moderate SW by increasing urine volume and urinary electrolyte excretion (Uex), whereas animals responded to high SW by either increasing Uex or altering drinking patterns to minimize salt stress.

## Introduction

Under tropical conditions and during periods of global warming, dairy animals are increasingly impacted by prolonged high ambient temperatures throughout the year [1]. The negative effects of prolonged high ambient temperature exposure could be characterized in part by altering either animal behavior, such as body temperature, respiratory rate, eating, and drinking patterns, or changing cellular stress responses, such as the expression of heat shock proteins (HSPs), including HSP60, HSP70, and HSP90 [2]. In addition to global warming, the Mekong River Delta area in Vietnam has a risk of salinity intrusion due to rising sea levels in recent years. Changes in sea level can affect coastal surface water and groundwater. The measured salinity levels in some rivers in the coastal provinces of the Mekong River Delta ranged from 0.6% to 1.5% of total dissolved salts (TDS) during 2016 [3].

Interestingly, crossbred dairy goats can be raised, and crossbred dairy goat farming is well-developed in the Mekong River Delta. Our previous studies have compared the breed specific for salinity water in goats between indigenous and crossbred meat or dairy types [3–6]. Crossbred Boer as meat-type goats increased water intake (WI) when challenged with 1.5% diluted seawater (SW) [3, 6], whereas crossbred Saanen as dairy-type goats decreased WI by 1.5% SW [5]. Growing Bach Thao goats as an indigenous breed apparently decreased their WI when tested with 1.5% SW [4, 6]. Moreover, Camels drank less saline water to decrease salt stress, whereas sheep and goats managed salt load by drinking saline water, excreting more urine, and increasing their filtration rate [7]. It has been demonstrated that the ability of small ruminants to tolerate various salt loads in drinking water is partly related to their kidney function [8]. A salt taste in mice can trigger two divergent behavioral responses: A low salt solution concentration can attract mice, whereas a high sodium chloride (NaCl) concentration is rejected by mice [9]. With high salt intake (4% of NaCl), vasopressin neurons of the paraventricular nucleus (PVN) of the hypothalamus are responsive [10]. It is well established that vasopressin or antidiuretic hormone (ADH) influences not only kidney function but also part of the stress response. The short-term effects of drinking 1.5% SW in crossbred dairy goats fed at a high ambient temperature affected water balance, thermoregulation capacity, and stress response in part through ADH [5]. In addition to the breed-specific preference for SW, we have previously demonstrated breed-specific delays in SW responses between crossbred dairy goats and indigenous Bach Thoa goats [5, 11]. Although crossbred lactating goats showed a significant decrease in daily WI within 2 weeks after 1.5 SW treatment [5], the onset of the effect of SW on daily WI in Bach Thoa goats was 5 weeks [11]. This information suggested that the control of daily WI in goats is apparently under two mechanisms.

This study aimed to investigate the prolonged effects of different SWs (from low to high salinity) in crossbred dairy goats on behavioral and physiological responses and water balance under tropical conditions. Our primary hypothesis was to investigate whether the delayed response in SW exists in crossbred dairy goats.

## Materials and Methods

### Ethical approval

The study was approved by the Animal Ethics and Welfare Committee, Can Tho University (#3559), Vietnam.

### Study period and location

The experiment was conducted from February to April 2020 at Experimental Farm, College of Rural Development, Can Tho University.

### Experimental design and animal care

The experiment was conducted on 20 crossbred dairy goats (Saanen × Bach Thao) for 90 days in milking, with an average body weight of 33.97 ± 1.00 kg, and a parity number was from 2 to 3 parity. All animals were kept in individual metabolic cages in 1.2 × 1.0 m shaped pens with wood floors under natural light (light on and off: 0600 and 1800 approximately) for 7 days of adaptation and 49 days (7 weeks) of the experimental period. Milking was performed by hand twice per day (07:00 and 14:00). Twenty dairy goats were selected and divided into four groups of five animals, each based on body weight and milk yield. Animals received either fresh drinking water (SW0.0, control) or diluted SW at concentrations of 0.5% (SW0.5, low salinity), 1% (SW1.0, moderate salinity), or 1.5% (SW1.5, high salinity). A high SW concentration of 1.5% was selected based on our previous studies [3–6]. The experiment used concentrated SW (9%) mixed with fresh water to achieve water with salt concentrations of 0.5%, 1%, and 1.5%, as described by Nguyen *et al*. [5]. Briefly, the final concentration at each level was calculated according to the formula C1V1 = C2V2 (where C1 is the concentration of the starting solution; V1 is the volume of the starting solution; C2 is the concentration of the final solution; and V2 is the volume of the final solution). The ambient temperature and humidity were used to calculate the temperature and humidity index (THI: Table-1), according to Nguyen *et al*. [3].

**Table-1:** Average environmental conditions during this experiment from daytime of the 1^st^ to 7^th^ week.

Time	Ambient temperature (°C)	Humidity (%)	THI
07:00	26.00 ± 1.00	75.50 ± 1.50	76.09 ± 1.39
09:00	28.00 ± 1.00	71.50 ± 3.50	78.67 ± 1.06
11:00	30.25 ± 0.25	62.50 ± 2.50	80.75 ± 0.74
13:00	31.50 ± 0.50	55.00 ± 1.00	81.30 ± 0.84
15:00	32.00 ± 1.00	65.50 ± 2.50	83.78 ± 1.88
17:00	29.25 ± 0.25	70.00 ± 2.00	80.39 ± 0.66
19:00	27.50 ± 0.50	73.50 ± 1.50	78.20 ± 0.96

Ambient temperature (°C), relative humidity (%), and temperature–humidity index (THI).

### Data collection

Feed intake was recorded daily throughout the experiment (from Day 1 to Day 49). Each goat was fed concentrate after the morning milking (08:00). The total amount of concentrate was calculated using milk yield, whereas water and natural grass were individually provided *ad libitum*. Feed and refusal samples from concentrate and natural grass were collected daily for daily dry matter intake (DMI). The eating patterns from each period were also measured from 08:00–14:00, 14:00–19:00, and 19:00–07:00 h on days 31 and 32 of the experiment. The eating pattern described above was used in the present experiment based on our previous findings that >70% of daily DMI intake is from daytime eating pattern [12]. As previously described by Nguyen *et al*. [4], feed samples were kept in the freezer (–20°C). At the end of the experiment, all feed samples were thawed and mixed thoroughly, and subsamples were dried at 65°C overnight for analysis of DM, Ash, crude protein, neutral detergent fiber, and acid detergent fiber. The chemical compositions of the experimental diets are presented in Table-2.

**Table-2:** Chemical composition of the experimental diets (DM basis).

Items (%)	Concentrate	Natural grass
DM	70.72	20.52
CP	15.12	7.61
ADF	27.91	44.32
NDF	48.48	62.50
Ash	9.92	9.67

CP=Crude protein, NDF=Neutral detergent fiber, ADF=Acid detergent fiber

Drinking water from either the freshwater (SW0.0) or SW group was provided to each goat daily with an individual open bucket. WI was recorded every day as daily drinking behavior from day 1 to day 49 of the experiment to measure the accurate behavioral data. WI was measured by subtracting the weight of water offered from the weight of water refusal. In addition, drinking patterns as the WI from each period were measured from 08:00 to 14:00, 14:00–19:00, and 19:00–07:00 h on days 31 and 32 of the experiment. All goats were weighed in the morning feeding on days 0 and 49 of the experiment. Rectal temperature (RT) and respiration rate (RR) were determined at 07:00, 09:00, 11:00, 13:00, 15:00, 17:00, and 19:00 h on day 30 of the experiment. RT was measured using a digital clinical thermometer (C202, Terumo, Tokyo, Japan. ±0.1°C). The RR was measured by counting the movement of the flank within 1 min.

On day 25, blood samples were collected at 07:00 h before morning feeding and then placed in a lithium heparin tube, kept in crushed ice, and centrifuged at 2500 × *g* for 10 min. The plasma samples were stored at −20°C until analysis. With the individual metabolic cage, total urine and feces can be separated and measured. Total urine and feces were recorded from days 25 to 29. The water balance from this experiment was calculated using the difference between water input (ingested water and feed water) and water output (milk, urinary excretion, and feces), without considering water metabolism. In addition, urine samples were collected at 50 mL, filtered through two layers of cheesecloth, and analyzed later. Plasma creatinine was determined using an automatic clinical chemistry analyzer (XL200, Erba Mannheim, Germany). Plasma HSP70 concentration was determined using an enzyme-linked immunosorbent assay kit specific for multispecies hormones (MBS738005, MyBioSource, San Diego, CA, USA). The sensitivity of the HSP70 assay was 0.1 ng/mL. The intra-assay variation in HSP70 measurement was 2.05%. Plasma and urine electrolytes were measured using an automatic analyzer (ST200 PRO, Sensa Core, India). In addition, this study also calculated urinary electrolyte excretion (Uex) by the following formula:

Urinary electrolyte excretion (Uex) = U_e_ × V

Where U_e_: urinary electrolyte concentration, V: Urine volume.

### Statistical analysis

The data are presented as the mean ± standard error of the mean. The commercial computer software GraphPad Prism 8.0 (GraphPad Software, Boston, Massachusetts, USA) was used for data analysis and scientific graphing. The data for DMI and WI by weeks were analyzed using a repeated two-way analysis of variance (ANOVA). The data for eating and drinking patterns, water balance, RT, RR, plasma, and urinary electrolytes were analyzed by a one-way ANOVA. The significance of the main effects or pairwise comparisons was assessed using Tukey’s honestly significant difference test. Significance was declared at p < 0.05. Data analysis was performed using a mixed linear model with treatment as the fixed effect and animals as the random effect. The mixed model used in this study is as follows:

y = μ + Ti + Dj(Ti) + Ak + eijkl,

Where y = dependent variable, μ = general mean, Ti = effect of treatment i, Dj(Ti) = effect of day in treatment (Ti), Ak = random effect of the animal, and eijk = residual error

## Results

### Effects of high salinity in drinking water on DMI and DMI patterns

**Figure-1:**
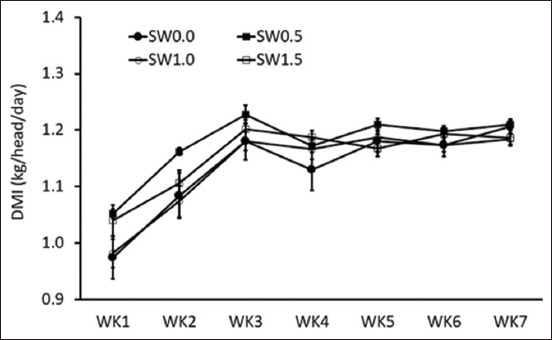
Effects of diluted seawater levels in drinking water on the average daily dry matter intake in lactating crossbred goats. SW0.0=Fresh water, SW0.5=Diluted seawater at 0.5%, SW1.0=Diluted seawater at 1.0%, SW1.5=Diluted seawater at 1.5%.

Throughout the experiment, the week had a significant effect based on the average daily DMI (Figure-1; p < 0.05). The average daily DMI can be divided into the adaptation period (weeks 1–2) and the plateau period (weeks 3–7). However, SW did not influence the daily DMI in drinking water among the treatment groups from both periods (Figure-1; p > 0.05). When analyzing the DMI pattern (Day 31 and 32 of the experiment), dairy goats consumed more feed in SW0.5 and SW1.0 than in SW1.5 from 14:00–19:00 h (Table-3, p < 0.01), whereas dairy goats in SW0.0 and SW1.5 from 19:00–07:00 h had a greater DMI than those in SW0.5 and SW1.0 (p < 0.01). As a result, the total daily DMI did not differ between treatments (Table-3; p > 0.05). Similarly, the percentage of total daily DMI patterns differed significantly between treatments from 14:00–07:00 h (Table-3; p < 0.01). Dairy goats in SW0.5 and SW1.0 had a higher percentage of total daily DMI from 14:00–19:00 h and then decreased from 19:00–07:00 h compared to SW0.0 and SW1.5. In contrast, there was no influence of treatment on DMI and DMI patterns from 08:00–14:00 h in this study. Moreover, there were no differences in the effects of SWs on body weight and milk yield.

**Table-3:** Effects of diluted seawater levels in drinking water on daily dry matter intake (DMI, g) and intake distribution (%) in lactating crossbred goats; means were calculated across experimental days 31 and 32 by daytime.

Items	Treatment				SEM	p-value

	SW0.0	SW0.5	SW1.0	SW1.5		
Total DMI (g/head/day)	1.233	1.274	1.292	1.234	0.02	0.21
08:00–14:00 (g/head)	0.602	0.651	0.672	0.654	0.02	0.20
14:00–19:00 (g/head)	0.526^ab^	0.585^a^	0.576^a^	0.455^b^	0.02	0.01
19:00–07:00 (g/head)	0.106^a^	0.037^b^	0.044^b^	0.125^a^	0.01	0.01
DMI (% total daily)						
08:00–14:00	48.80	51.04	51.90	52.87	1.21	0.15
14:00–19:00	42.65^ab^	46.00^a^	44.71^a^	36.917^b^	1.74	0.01
19:00–07:00	8.55^a^	2.96^b^	3.39^b^	10.22^a^	1.25	0.001

SW0.0=Fresh water, SW0.5=Diluted seawater at 0.5%, SW1.0=Diluted seawater at 1.0%, SW1.5=Diluted seawater at 1.5%, SEM=Standard error of the mean. ^a-b^means with different superscripts in the same row differ significantly (p < 0.05)

### Effects of high salinity in drinking water on WI pattern, water balance, and physiological responses

**Figure-2:**
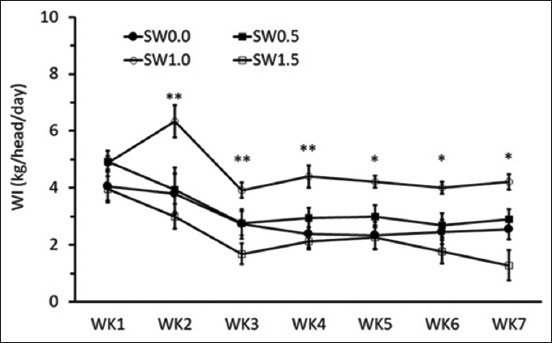
Effects of diluted seawater levels in drinking water on the average daily water intake (WI) in lactating goats. Asterisks indicate significant differences between treatments by weeks. *p < 0.05; **p < 0.01. SW0.0=Fresh water, SW0.5=Diluted seawater at 0.5%, SW1.0=Diluted seawater at 1.0%, SW1.5=Diluted seawater at 1.5%.

Similar to the effect of treatment protocol on the average daily DMI, the average daily WI contained two periods, including adaptation and plateau periods. Different SW concentrations significantly influenced the amount of daily WI. From weeks 2 to 7 of the experimental period, goats from SW1.0 appeared to have higher daily WI than the other groups (Figure-2; p < 0.05). In addition, this study also determined the WI pattern during the day and night during days 31 and 32 of the experiment. The current results showed that during daytime (08:00–19:00 h), dairy goats in SW1.0 had increased WI compared with those in SW0.0 and SW1.5 (Table-4; p < 0.05), whereas WI was similar among treatments during night time (19:00–07:00 h: Table-4; p > 0.05). When analyzing %, WI as % total daily from SW1.5 was lower than the control group (SW0.0) from 08.00–14.00 h, but %WI from SW1.5 was higher than the control group (SW0.0) from 14.00–19.00 h (Table-4, p < 0.05).

**Table-4:** Effects of diluted seawater levels in drinking water on daily water intake (kg) and intake distribution (%) in lactating crossbred goats, means across experimental days 31 and 32.

Items	Treatment				SEM	p-value

	SW0.0	SW0.5	SW1.0	SW1.5		
Total WI (kg/head/day)	3.29^ab^	5.64^ab^	6.70^a^	1.79^b^	1.09	0.02
08:00–14:00 (kg/head)	2.94^ab^	4.49^ab^	5.36^a^	1.30^b^	0.89	0.03
14:00–19:00 (kg/head)	0.012^b^	0.350^ab^	1.180^a^	0.300^ab^	0.27	0.04
19:00–07:00 (kg/head)	0.338	0.800	0.160	0.190	0.18	0.08
WI (% total daily)						
08:00–14:00	90.95^a^	77.86^ab^	83.10^ab^	64.54^b^	5.79	0.03
14:00–19:00	0.23^b^	8.33^ab^	14.28^ab^	25.24^a^	4.93	0.02
19:00–07:00	8.82	13.81	2.62	10.22	3.09	0.12

SW0.0=Fresh water, SW0.5=Diluted seawater at 0.5%, SW1.0=Diluted seawater at 1.0%, SW1.5=Diluted seawater at 1.5%, SEM=Standard error of the mean. ^a-b^means with different superscripts in the same row differ significantly (p < 0.05)

The water balance data investigated during days 25–29 of the experimental period are presented in Table-5. SW did not affect WI from feed and water excretion through milk and feces (p > 0.05). Importantly, urine excretion was significantly higher in the SW1.0 group than in the SW1.5 group. Overall, the lowest water balance calculated for the SW1.5 group was significantly lower than that in the control (SW0.0) group. The plasma ADH concentration in SW1.5 was higher than in the other groups; however, a significantly higher concentration was observed between SW1.5 and SW0.0 (Table-5, p < 0.05). The effect of SW on RT was prominent in the afternoon (Table-6). From 07:00–13:00 h, RT did not differ among treatments, but RT in the high saline group (SW1.5) was higher than that in the low saline group (SW0.5 and SW1.0) from 15:00–17:00 h. The RR with SW1.5 was higher than that with the other treatments throughout the daytime, particularly between SW1.5 and SW0.0 (Table-6, p < 0.05).

**Table-5:** Effects of diluted seawater levels on water balance and plasma ADH concentrations in lactating crossbred goats.

Items	Treatment				SEM	p-value

	SW0.0	SW0.5	SW1.0	SW1.5		
Water intake from drinking water (kg/day)	2.90^b^	3.34^ab^	4.63^a^	2.27^b^	0.36	0.002
Water intake from feed (kg/day)	2.33	2.43	2.25	2.32	0.07	0.43
Urinary excretion (kg/day)	1.91^b^	2.93^ab^	3.48^a^	2.14^b^	0.25	0.01
Water excretion via milk (kg/day)	0.49	0.53	0.60	0.46	0.07	0.51
Water excretion via feces (kg/day)	0.69	0.50	0.62	0.71	0.06	0.14
Water balance (kg/day)	0.96^a^	0.58^ab^	0.79^ab^	0.08^b^	0.20	0.03
ADH (pg/mL)	8.52^b^	13.00^ab^	11.18^ab^	16.80^a^	1.86	0.04

SW0.0=Fresh water, SW0.5=Diluted seawater at 0.5%, SW1.0=Diluted seawater at 1.0%, SW1.5=Diluted seawater at 1.5%, ADH=Antidiuretic hormone, SEM=Standard error of the mean. ^a-c^means with different superscripts in the same row differ significantly (p < 0.05)

**Table-6:** Effects of diluted seawater levels on rectal temperature and respiration rate during daytime in lactating crossbred goats.

Time	Treatment				SEM	p-value

	SW0.0	SW0.5	SW1.0	SW1.5		
Rectal temperature (°C)						
07:00	39.1	39.0	39.1	39.0	0.1	0.93
09:00	39.2	39.2	39.4	38.9	0.2	0.26
11:00	39.2	38.4	39.5	39.1	0.2	0.13
13:00	39.2	38.6	39.0	39.2	0.2	0.18
15:00	39.3^ab^	38.6^b^	38.9^ab^	39.6^a^	0.2	0.01
17:00	39.3^a^	38.8^b^	38.8^b^	39.5^a^	0.1	0.001
19:00	39.2	39.0	39.3	39.4	0.1	0.16
Respiration rate (breath/min)						
07:00	42^b^	40^b^	30^b^	69^a^	5	0.001
09:00	38	50	63	56	7	0.15
11:00	42	52	51	61	8	0.36
13:00	42	54	50	63	5	0.06
15:00	46	50	53	69	6	0.09
17:00	54^b^	51^b^	69^ab^	74^b^	5	0.01
19:00	57	53	60	73	7	0.22

SW0.0=Fresh water, SW0.5=Diluted seawater at 0.5%, SW1.0=Diluted seawater at 1.0%, SW1.5=Diluted seawater at 1.5%, SEM=Standard error of the mean. ^a-b^means with different superscripts in the same row differ significantly (p < 0.05)

### Effects of high salinity in drinking water on plasma electrolytes, plasma HSP70 concentration, urinary electrolyte concentration, and excretion

The concentrations of electrolytes and creatinine levels in blood did not differ among treatments (Table-7, p > 0.05). Similarly, the plasma HSP70 concentration did not differ significantly among the treatments. In this study, dairy goats in SW1.0 had a higher urine volume than those in the other treatments, particularly between SW1.0 and SW0.0 and SW1.5 (Table-8, p < 0.01). The urinary Na^+^ and K^+^ concentrations in SW1.5 and SW1.0 were higher than those in the other treatments (Table-8, p < 0.01), whereas the urinary Cl level was similar among the treatments. The Uex of Na^+^ from SW1.5 and K^+^, Cl^-^ from SW1.0 was higher than that from SW0.0 and SW0.5 (Table-8, p < 0.01).

**Table-7:** Effects of diluted seawater levels in drinking water on plasma electrolyte, creatinine, and HSP70 concentrations in lactating crossbred goats.

Items	Treatment				SEM	p-value

	SW0.0	SW0.5	SW1.0	SW1.5		
Sodium (mmol/L)	146.34	148.92	146.46	146.82	0.87	0.17
Potassium (mmol/L)	4.01	3.72	3.67	3.92	0.19	0.55
Chloride (mmol/L)	107.9	109.0	109.4	109.3	0.60	0.32
Creatinine (µmol/L)	68.11	67.31	67.01	68.74	3.14	0.98
HSP70 (ng/mL)	16.00	17.95	18.97	17.66	1.14	0.36

SW0.0=Fresh water, SW0.5=Diluted seawater at 0.5%, SW1.0=Diluted seawater at 1.0%, SW1.5=Diluted seawater at 1.5%

**Table-8:** Effects of diluted seawater levels in drinking water on urine volume, concentration, and urinary electrolyte excretion in lactating crossbred goats (during experimental days 25–29).

Items	Treatment				SEM	p-value

	SW0.0	SW0.5	SW1.0	SW1.5		
UV (L/head/day)	1.91^b^	2.93^ab^	3.48^a^	2.14^b^	0.25	0.01
Urinary electrolytes concentration (mmol/L)						
Sodium	108.56^ab^	90.26^b^	73.92^b^	146.74^a^	13.64	0.01
Potassium	126.98^ab^	138.62^a^	150.91^a^	80.28^b^	12.24	0.01
Chloride	153.70	195.60	182.50	175.60	10.09	0.06
Urinary electrolytes excretion (mmol/head/day)						
Sodium	212.9^b^	189.9^b^	256.5^ab^	425.6^a^	45.60	0.01
Potassium	242.5^b^	301.9^b^	526.2^a^	247.9^b^	51.47	0.01
Chloride	295.6^b^	422.8^ab^	641.6^a^	515.3^ab^	60.61	0.01

SW0.0=Fresh water, SW0.5=Diluted seawater at 0.5%, SW1.0=Diluted seawater at 1.0%, SW1.5=Diluted seawater at 1.5%, SEM=Standard error of the mean. ^a-b^means with different superscripts in the same row significantly differ (p < 0.05)

## Discussion

In this study, lactating crossbred goats adapted to low-and-moderate SW (SW0.5 and SW1.0) by increasing their drinking behaviors. The increased WI enhanced kidney function by increasing urine volume and elevating Uex. For high SW (SW1.5), drinking behavior decreased to minimize salt stress. Despite a low urine volume, the goats responded to high SW by increasing Uex. These responses corresponded to the lowest water balance in the high SW group and higher RT and RR.

There were no significant effects of diluted SW on DMI throughout the experiment. Similar findings were reported in goats [13] and sheep [14]. Peirce [15] found that feed intake in sheep was not affected by SW at concentrations ranging from 1.3% to 1.5%; however, feed intake sharply decreased when animals drank 2% saline water. However, some studies [3, 16, 17] on Holstein steers and Boer goats found that DMI increased in the low-saline water group at the beginning of the experiment, which differed from the responses in the present study. In this study, we also determined the effect of high SW on the DMI pattern. Although DMI was not affected by diluted SW levels, there was a significant difference in DMI from 14:00–19:00 h (afternoon feeding) and from 19:00–07:00 h (night time). Animals in SW1.5 ate less DMI during afternoon feeding and consumed more than other treatments to compensate for DMI at night. The same tendency was observed when the DMI was presented as a percentage of the total daily DMI (%). These results indicate that dairy goats adjusted their DMI pattern when they drank different levels of diluted SW according to the day and night cycles from the current experiment.

SW affected daily WI, starting from the 2^nd^ to 7^th^ weeks in this study (Figure-2). Lactating goats increased WI in SW at concentrations of 0.5%–1%, but animals decreased WI at 1.5% SW. Similarly, a previous study by Nguyen *et al*. [4] found that WI was greater at low salinity levels, but water consumption decreased when the saline level increased up to 2%. However, Mohammed [18] reported that Nubian goats fed 1.5% NaCl had increased WI compared to those fed fresh water. Similarly, Nguyen *et al*. [3] and Nguyen and Thammacharoen [6] reported higher WI in Boer crossbred goats with SW (1.5% TDS). The results from the current study indicate breed specific for the response of salinity level in drinking water in goats. The meat-type breed (Boer and Nubian) increases WI and the dairy-type (Saanen) decreases WI during challenges with SW1.5 [3, 5, 6, 18]. Moreover, in the present experiment, lactating goats consumed more water at low or moderate levels of diluted SW (1.0%), but at high salinity, SW (1.5%) decreased WI as part of an aversive response [9] to salt stress. This partially indicates the preference for salinity levels in the drinking water of dairy goats and suggests that a specific preference test [4] should also be performed in crossbred Saanen. Another important behavioral response that suggested breed specificity was the onset of drinking SW, which occurred faster (within 1 week) than our previous native breed [11]. The difference in daily WI between the moderate SW and other treatments from the current experiment mainly originated from 08:00–19:00, whereas the WI during nighttime was similar among the treatments (Table-4). Interestingly, the daily WI between the fresh and high SW groups was not significantly different throughout the study. This may be due to the dairy goats used in this experiment trying to prevent salt stress from high SW. In addition, the animals from this study also adapted with high SW by distributing their WI throughout the day (Table-4). Normally, animals consume a high percentage of water after the main meal, which was confirmed by the present study when animals drank >70% of daily WI in the morning feeding and then gradually decreased in the next meal. However, this phenomenon differed from the high-SW group; the animals drank <10–15% compared to the low-to-moderate SW group in the morning feeding and then had a higher ratio of WI during the afternoon feeding (Table-4). Moreover, another possible factor is that dairy goats can cope with saline water when they can compensate for ingested salt with water from their feed, that is, natural grass.

The present results indicate that lactating crossbred goats consumed saline concentrations of 0.0%–1.0%, which increased WI and urinary excretion. In contrast, WI decreased and urine volume remained unchanged at the 1.5% saline concentration compared with the freshwater group. Therefore, the water balance decreased as saline levels increased in this study. Interestingly, WI and urine volume from SW1.5 were similar to freshwater (0.0%), but the water balance from SW1.5 was significantly lower than that from SW0.0. As a result, the plasma ADH concentration in SW1.5 was greater than that in the other treatments in this experiment. The results for water balance from 0.0% to 1.0% saline levels agreed with previous studies on feedlot lambs [19]. In contrast, Assad and Elsherif [20] found that blood volume, plasma volume, and extracellular fluid content decreased with increasing water salinity for ewes, similar to the result for SW1.5 in this study.

Chaiyabutr *et al*. [21] and Nguyen *et al*. [22] reported that a higher water balance provides a reservoir of soluble metabolites for milk synthesis and is also useful for slowing down body temperature elevation during high ambient temperatures in dairy cows and dairy goats. This would be confirmed by the current experiment when the water balance was higher in SWs and the RT was lower than in SW1.5 at 15:00–17:00 h. Moreover, higher RTs from high saline intake may be due to greater heat production for mineral urinary excretion, as suggested by Arieli *et al*. [23]. Previous studies by Nguyen [4] and Mdletshe *et al*. [24] reported that RT was increased by saline water. In contrast, RTs from other time points in this experiment did not differ among treatments.

Similarly, Mdletshe *et al*. [24] found that a saline water content of 0.55%–1.1% did not affect the RT of goats. The results of the present study show that dairy crossbred goats had increased RR in relation to increments in ambient temperature and THI throughout the day. However, the animal responses at high ambient temperatures were not similar to those of the other treatments. Animals from SW1.5 had increased RRs from 13:00–17:00 h (THI from 80.39 to 83.78), even when heat stress was absent at 07:00 h (THI at 07.00 h was 76.09 ± 1.39) compared with the other treatments. A similar finding was reported by Mdletshe *et al*. [24]. However, Runa *et al*. [17] found that the RR of Boer goats was unaffected by saline content when animals were exposed to temperate conditions.

This study showed that plasma electrolytes did not affect water salinity among the groups and were within the normal ranges for healthy goats, as mentioned by Zoidis and Hadjigeorgiou [25] and Runa *et al*. [26]. Previous studies by Nguyen *et al*. [3, 4] have found that plasma Na^+^ levels were in the normal range after goats drank saline water for 4 or 2 weeks. The current study shows that goats drinking low to high-saline water were still in the reference range and maintained constant plasma Na^+^ and Cl^-^ concentrations. In sheep, the ability to tolerate varying salt contents in drinking water has been linked to kidney function [8]. This ability of sheep may be due to renal adjustments that increased filtration and eliminated salt. Therefore, the urine volume, urinary electrolyte concentration, and excretion were affected by SW, particularly in the SW1.0, SW1.5, and SW0.0 groups.

Creatinine is a byproduct of muscle metabolism and is freely excreted by glomerular filtration. Thus, creatinine can be used as an indicator of renal function [27]. In the present study, the creatinine concentration in plasma remained unchanged among the treatments and was at the reference level [27], indicating no adverse effects on kidney function due to the consumption of SW by goats. This result is similar to a previous study by Zoidis and Hadjigeorgiou [25], in which goats drank high-saline water without affecting plasma creatinine levels, whereas plasma creatinine levels increased with high saline WI [25].

In the current study, dairy goats fed highly diluted SW may undergo osmotic stress, as suggested by Zoidis and Hadjigeorgiou [25]. In addition, dairy goats that drank 1.5% diluted SW from the current study also had increased plasma ADH concentrations. Mitchell *et al*. [10] suggested that mice drinking 4% saline water, not 2% saline water, were likely hypovolemic and hyperosmotic due to reduced fluid intake and consumption of hypertonic solutions. This phenomenon partially contributed to the increase in PVN-amygdala signaling by stress neuropeptides. The different responses between this study and previous studies on saline water to VP levels may be due to different species and saline water levels related to osmotic stressors. In the present experiment, the plasma HSP70 concentration are comparable with that reported by Yamani and Koluman [28] and was not affected by different levels of diluted SW in drinking water. This result indicates that SW did not increase HSP70 levels in crossbred dairy goats within the salinity range. Moreover, the animals in the present study adjusted their adaptation by altering either their eating and drinking patterns or urinary excretion to minimize salt stress.

## Conclusion

Lactating crossbred goats adapted with low-and-moderate salinity levels in drinking water (SW0.5 and 1.0) by increasing drinking and urine volume and having higher Uex. In contrast, animals responded to high salinity levels in drinking water (SW1.5) either by increasing Uex or decreasing total daily WI and altering drinking patterns. The rapid behavioral response to SW was also demonstrated. Moreover, high salinity levels in drinking water may activate the osmotic control system by increasing plasma ADH concentration. The total daily DMI was not affected by SW, but animals from the high-SW group shifted their eating patterns to adapt to high SW. In addition, water balance was decreased in the high SW group. This appears to be an important factor that caused the decrease in thermoregulation capacity through higher RT and RR in the high saline group.

## Authors’ Contributions

NT: Conceptualization, methodology, formal analysis, investigation, data curation, and drafted and revised the manuscript. KVT, KKTN, and NTN: Investigation and data curation. ST: Formal analysis, data curation and drafted and revised the manuscript. NC: Conceptualization and revised the manuscript. All authors have read and approved the final manuscript.
